# Total Serum Cholesterol and Cancer Incidence in the Metabolic Syndrome and Cancer Project (Me-Can)

**DOI:** 10.1371/journal.pone.0054242

**Published:** 2013-01-23

**Authors:** Susanne Strohmaier, Michael Edlinger, Jonas Manjer, Tanja Stocks, Tone Bjørge, Wegene Borena, Christel Häggström, Anders Engeland, Gabriele Nagel, Martin Almquist, Randi Selmer, Steinar Tretli, Hans Concin, Göran Hallmans, Håkan Jonsson, Pär Stattin, Hanno Ulmer

**Affiliations:** 1 Department of Medical Statistics, Informatics and Health Economics, Innsbruck Medical University, Innsbruck, Austria; 2 Department of Surgery, Malmö University Hospital, Lund University, Malmö, Sweden; 3 Department of Surgical and Perioperative Sciences, Urology and Andrology, Umeå University, Umeå, Sweden; 4 Institute of Preventive Medicine, Copenhagen University Hospital, Copenhagen, Denmark; 5 Department of Public Health and Primary Health Care, University of Bergen, Bergen, Norway; 6 Norwegian Institute of Public Health, Oslo/Bergen, Norway; 7 Institute of Epidemiology and Medical Biometry, Ulm University, Ulm, Germany; 8 Agency for Preventive and Social Medicine, Bregenz, Austria; 9 Department of Surgery, Skåne University Hospital Lund and Lund University, Lund, Sweden; 10 Cancer Registry of Norway, Institute of Population-based Cancer Research, Montebello, Oslo, Norway; 11 Department of Public Health and Clinical Medicine, Nutritional Research, Umeå University, Umeå, Sweden; 12 Department of Radiation Sciences, Oncology, Umeå University, Umeå, Sweden; 13 Urology Service, Department of Surgery, Memorial Sloan-Kettering Cancer Center, New York, New York, United States of America; Sookmyung Women’s University, Republic of Korea

## Abstract

**Objective:**

To investigate the association between total serum cholesterol (TSC) and cancer incidence in the Metabolic syndrome and Cancer project (Me-Can).

**Methods:**

Me-Can consists of seven cohorts from Norway, Austria, and Sweden including 289,273 male and 288,057 female participants prospectively followed up for cancer incidence (n = 38,978) with a mean follow-up of 11.7 years. Cox regression models with age as the underlying time metric were used to estimate hazard ratios (HR) and their 95% confidence intervals (CI) for quintiles of cholesterol levels and per 1 mmol/l, adjusting for age at first measurement, body mass index (BMI), and smoking status. Estimates were corrected for regression dilution bias. Furthermore, we performed lag time analyses, excluding different times of follow-up, in order to check for reverse causation.

**Results:**

In men, compared with the 1st quintile, TSC concentrations in the 5th quintile were borderline significantly associated with decreasing risk of total cancer (HR = 0.94; 95%CI: 0.88, 1.00). Significant inverse associations were observed for cancers of the liver/intrahepatic bile duct (HR = 0.14; 95%CI: 0.07, 0.29), pancreas cancer (HR = 0.52, 95% CI: 0.33, 0.81), non-melanoma of skin (HR = 0.67; 95%CI: 0.46, 0.95), and cancers of the lymph−/hematopoietic tissue (HR = 0.68, 95%CI: 0.54, 0.87). In women, hazard ratios for the 5th quintile were associated with decreasing risk of total cancer (HR = 0.86, 95%CI: 0.79, 0.93) and for cancers of the gallbladder (HR = 0.23, 95%CI: 0.08, 0.62), breast (HR = 0.70, 95%CI: 0.61, 0.81), melanoma of skin (HR = 0.61, 95%CI: 0.42, 0.88), and cancers of the lymph−/hematopoietic tissue (HR = 0.61, 95%CI: 0.44, 0.83).

**Conclusion:**

TSC was negatively associated with risk of cancer overall in females and risk of cancer at several sites in both males and females. In lag time analyses some associations persisted, suggesting that for these cancer sites reverse causation did not apply.

## Introduction

Since the 1980s several epidemiological studies have reported an association between higher total serum cholesterol (TSC) levels and lower overall or site-specific cancer incidence and mortality [1]–[9], whereas others found higher cancer risk in people with high TSC levels [10]–[13], no significant relation [14]–[18], or a U-shaped association, that is both low and high TSC levels being significantly associated with increased cancer risk [19].

It has been suggested that the observed inverse associations have to be attributed to an effect of preclinical cancer or disease on cholesterol levels (i.e. metabolic depression or increased utilization of cholesterol during carcinogenesis [20]) rather than reflecting a true causal relationship. The hypothesis of reverse causation is strongly supported by a recent Mendelian randomization study [21] and by the observation that the inverse associations between high cholesterol levels and cancer risk and mortality weakened or even disappeared when the first few years of study follow-up were excluded [1], [9], [22]. However, some studies found inverse associations with time lags of 4 or even more years between baseline cholesterol level and cancer diagnosis [7], [20], [23], so the possibility that there may be a direct effect of low cholesterol on cancer can still not be completely ruled out.

More recent studies [24], [25] on cholesterol and cancer incidence, including partly data also used in this study [25], added additional evidence for the reverse causation hypothesis again. Nevertheless, relatively modest sample sizes and differences in study populations, length of follow-up, study endpoints and statistical procedures may all have contributed to the lack of consistency in results of previous studies.

Assessment of cholesterol levels on a single occasion results in a substantial random error due to variability in the measurement process or real but short-term biological variability. Such inaccuracy in exposure measurement may lead to underestimation [26] of the risk factor outcome association through the so called regression dilution bias. Prospective studies on metabolic factors and risk of cardiovascular disease, which utilized repeated exposure measurement to apply methods to correct for regression dilution bias, presented stronger associations than those based on single baseline measured exposures [27]–[29].

Motivated by the inconsistency in the literature and the failure to account for regression dilution, the aim of this study was to investigate the association between TSC and the overall and site-specific cancer incidence in a large study population containing seven European cohorts.

## Materials and Methods

### Study Population

The Metabolic syndrome and Cancer project (Me-Can) includes data from population-based cohorts from Norway, Austria, and Sweden, and aims at investigating associations between metabolic factors and cancer risk.

A detailed description of the project has been published previously [30]. In 2006, data from seven existing cohorts from Norway (Oslo study, Norwegian Counties study, the Cohort of Norway, Age 40 programme), Austria (Vorarlberg Health Monitoring and Prevention Programme), and Sweden (Västerbotten Intervention Project, Malmö Preventive Project) were pooled. Participants in the cohorts had undergone one or more health examinations between 1972 and 2005, and information on lifestyle and socio-demographic factors had been recorded and available data have been managed accordingly. For the present study information on age, weight, height, TSC level, and smoking status of 289,273 men and 288,057 women was used.

### Measurements

Anthropometric measurements were conducted in a similar way in all Me-Can cohorts, with participants wearing light indoor clothes and no shoes. Regarding smoking habits, participants were asked to fill in a questionnaire except in VHM&PP where respective questions were asked by the examining physician and the information was entered directly into a database. Participants were classified as never, former, and current smokers.

Fasting time before blood was drawn varied across the different cohorts [30]. In the Norwegian cohorts, fasting was not required before the examination, and fasting time was recorded as less than 1, 1–2, 2–4, 4–8, or more than 8 hours. Fasting time in Västerbotten Intervention Project was recorded as less than 4, 4–8, or more than 8 hours, and from 1992 onwards, participants were asked to fast for at least 8 hours before the examination. In Malmö Preventive Project and, after the initial 3 years, in the Austrian programme, a minimum of 8 hours of fasting was used as the standard procedure. For the analyses, information on fasting status was summarized into the categories of less than 4, 4–8, and more than 8 hours.

In the Oslo and the Norwegian Counties study serum levels of total cholesterol were measured applying a non-enzymatic method, whereas in all other cohorts an enzymatic method was used. Measurements obtained by a non-enzymatic method have been transformed according to 0.92×(cholesterol level) - 0.03 and are presented in mmol/l [30].

### Identification of Cases and Cohort Follow-up

Incident cancer cases were identified through linkages with national cancer registries of the respective countries and categorized according to the International Classification of Diseases, seventh revision. Follow-up ended at the date of the first primary cancer diagnosis, emigration, death or December 31, 2003 (in Austria), 2005 (in Norway), or 2006 (in Sweden), whichever occurred first.

### Statistical Analysis

Cox proportional hazard regression analyses were applied for men and women separately to investigate the association between TSC levels and site-specific cancer incidence. Subjects were followed until the date of first cancer diagnosis or were censored as described above. When analyzing a specific cancer site, this site was regarded as an event whereas all other sites were censored. Hazard ratios (HR) and respective 95% confidence intervals (CI) were estimated for TSC levels in quintiles (with cut-off levels determined separately for each sex, cohort, and fasting time category) and as a continuous variable (HRs per 1 mmol/l increment).

Age was utilized as the underlying time metric and all estimates were stratified by cohort, fasting time, and categories of birth year (before 1929, 1930–1939, 1940–1949, 1950–1959, 1960–1969, 1970 and later). Additionally, all analyses included adjustment for age at baseline (continuous), body mass index (BMI categories <22.5, 22.5–<25.0, 25.0–<27.5, 27.5–<30.0, 30.0–<32.5, ≥32.5- kg/m2), and smoking status (categories never, former, current smoker). Linear tests for trend were performed including TSC quintiles as an ordinal variable.

The proportionality assumption was checked applying a test based on Schoenfeld residuals. For some cancer sites there was an indication of violation of the proportionality assumption for BMI or smoking status. Additional models stratified for the respective variable were fitted, however estimates of hazard ratios for TSC did not change markedly. To check for reverse causation, various lag-time analyses were carried out, leaving out the first year, the first 5 years, and the first 10 years of follow-up.

In the main analyses hazard ratios were corrected for random error in TSC measurements using a method involving calculation of regression dilution ratio (RDR), similar to that described by Wood et al. [31]. Additionally, uncorrected hazard ratios were calculated and presented in supplement tables. Calculation of RDRs was based on data from subjects for whom two or more observations with the same fasting time before measurement were available; in total, data from 133,820 subjects and 406,364 health examinations were available. Altogether, the mean time between the baseline and the repeated measurement was 6.9 years (standard deviation [SD] = 3.9). Linear mixed effects models, treating the repeated measurements as the dependent variable and the baseline measurements as the independent variable and further including age at baseline, fasting time, smoking status, sex, birth year, BMI, and time since date of baseline examination as fixed effects and cohort as a random effect, were fitted. RDRs were estimated as the predicted regression coefficient at the time point six years after baseline measurement, i.e. at half the follow-up time. The obtained RDRs for TSC were 0.644 in men and 0.660 in women. Correction of hazard ratios was achieved by calculating exp (ln (HR)/RDR).

To assess whether statin prescription had an effect on the association between TSC and cancer incidence, additional analyses were performed with only baseline measurements that had been obtained before 1994. This timepoint was selected as the Scandinavian Simvastatin Study published in 1994 [32] was regarded as the starting point for the afterwards steadily increasing statin prescription.

Statistical analyses were performed with Stata (version 10, StataCorp LP, College Station, Texas) and R (version 2.7.2, used for RDR calculation). Two-sided P values lower than 0.05 were considered statistically significant.

### Ethics

The study was approved by The Research Review Board of Umeå, Sweden, the Regional Committee for Medical and Health Research Ethics, Southeast Norway and the Ethikommission of the Land Vorarlberg, Austria. Participants from Sweden and Austria provided written informed consent to participate in this study. In Norway, the participants were invited to come to the health survey and a questionnaire was sent together with the invitation. An attendance to the health examination where the participants delivered their filled in questionnaire, has been accepted by the Data Inspectorate as an informed consent, but not a written consent. Written consent was obtained from 1994.

## Results

### Baseline Characteristics

In Table 1 baseline characteristics of the study population are presented by cohort. Mean age at baseline varied between 40.3 (SD = 7.0) years in the NCS cohort and 47.5 (15.0) years in CONOR. BMI was highest in the Oslo cohort 26.6 (2.9) and lowest in NCS and MPP. The highest rate of people suffering from hypercholesterolaemia (TSC>6.2 mmol/l) was observed in the Oslo cohort with 50.2%, the lowest in the 40-y cohort with 23.4%. Mean follow-up ranged from 26.0 (8.0) years in the Oslo cohort to 6.1 (2.4) years in CONOR.

**Table 1:** Baseline characteristics of study participants in the Metabolic Syndrome and Cancer Project.

	Oslo	NCS	CONOR	40-y	VHM&PP	VIP	MPP
Inclusion period	1972–1973	1974–1983	1995–2003	1994–1999	1988–2002	1985–2005	1974–1992
Number (%)	16,760 (2.90)	51,024 (8.84)	109,868 (19.03)	128,887 (22.32)	159,280 (27.59)	78,818 (13.65)	32,693 (5.66)
Baseline age, years (mean (SD))	44.1 (5.6)	40.3 (7.0)	47.5 (15.0)	41.5 (1.9)	42.7 (15.4)	45.6 (9.6)	45.6 (7.4)
Smoking status, (n(%))							
Never smoker	3,303 (19.7)	18,022 (35.3)	48,859 (44.5)	24,042 (18.7)	105,328 (66.1)	45,756 (58.1)	12,170 (37.2)
Ex-smoker	3,975 (23.7)	9,185 (18.0)	28,255 (25.7)	80,959 (42.8)	13,960 (8.8)	16,292 (20.7)	5,882 (18.0)
Current smoker	9,482 (56.6)	23,817 (46.7)	32,754 (29.8)	23,886 (18.5)	39,992 (25.1)	15,291 (19.4)	14,589 (44.6)
Missing	0	0	0	0	0	1,479 (1.8)	52 (0.2)
BMI, kg/m^2^ (mean (SD))	26.6 (2.9)	24.6 (3.5)	26.1 (4.1)	25.5 (3.8)	24.8 (4.2)	25.8 (4.0)	24.6 (3.6)
Cholesterol, mmol/l, (mean(SD))	6.33 (1.19)	6.21 (1.26)	5.70 (1.18)	5.54 (1.01)	5.55 (1.19)	5.64 (1.19)	5.66 (1.08)
Quintiles, (mean (SD))							
1	4.83 (0.47)	4.66 (0.42)	4.26 (0.41)	4.28 (0.40)	4.06 (0.41)	4.12 (0.49)	4.29 (0.40)
2	5.75 (0.18)	5.51 (0.20)	5.03 (0.20)	4.99 (0.23)	4.88 (0.18)	5.00 (0.20)	5.07 (0.18)
3	6.33 (0.16)	6.11 (0.20)	5.61 (0.19)	5.47 (0.25)	5.46 (0.18)	5.71 (0.20)	5.60 (0.17)
4	6.92 (0.19)	6.77 (0.26)	6.26 (0.23)	6.00 (0.30)	6.10 (0.22)	6.19 (0.25)	6.16 (0.21)
5	8.08 (0.83)	8.05 (0.98)	7.46 (0.74)	7.01 (0.73)	7.32 (0.82)	7.39 (0.79)	7.24 (0.76)
Hypercholesterolemia, (n (%)) >6.2 mmol/l	8,409 (50.2)	23,575 (46.2)	33,101 (30.1)	30,178 (23.4)	42,611 (26.8)	22,674 (28.8)	9,244 (28.3)
Fasting							
<4 h	13,642 (81.4)	39,800 (78.0)	85,533 (77.9)	101,142 (78.5)	0 (0.0)	2,680 (3.4)	0 (0.0)
4 h–8 h	1,700 (10.1)	9,831 (19.27)	18,208 (16.57)	22,172 (17.20)	0 (0.0)	5,601 (7.1)	0 (0.0)
>8 h	1,418 (8.5)	1,393 (2.7)	6,127 (5.6)	5,573 (4.3)	159,280 (100.0)	70,537 (89.5)	32,693 (100.0)
Measurement method	Non- enzymatic	Non- enzymatic,Enzymatic from year 1980	Enzymatic	Enzymatic	Enzymatic	Enzymatic	Enzymatic
Follow-up, years (mean (SD))	26.0 (8.0)	25.8 (6.2)	6.1 (2.4)	7.4 (1.6)	10.4 (4.5)	7.5 (4.2)	21.1 (6.3)
Categories, (n)							
<1	69	183	902	364	458	6600	190
1–<5	447	1,087	42,663	2,083	27,978	23,381	1,035
5–<10	659	1,411	67,205	123,035	37,354	30,581	1,353
10–	15,654	48,546	0	3,769	93,948	24,856	30,305
Cancer cases, (n)	4,382	7,916	4,610	2,714	8,524	4,163	6,639

Abbreviations: BMI, body mass index; CONOR, Cohort of Norway; MPP, Malmö Preventive Project; NCS, Norwegian Counties Study; Oslo, Oslo study I; SD, standard deviation; VHM&PP, Vorarlberg Heath Monitoring and Prevention Programme; VIP, Västerbotten Intervention Project; 40-y, Age 40-programme.

### TSC and Risk of Incident Cancer

Hazard ratios corrected for regression dilution bias for total and site-specific cancer incidence by quintiles and per unit increment of TSC are presented in Table 2 and Table 3 for men and women respectively. In addition, uncorrected estimates can be found in Tables S1 and Tables S2.

**Table 2:** RDR corrected hazard ratios^a^ of incident cancer by cholesterol in quintiles (compared to the lowest quintile) and per unit increment in men.

		Quintile											
Site (ICD-7 code)		2		3		4		5			per 1 unit		(mmol/l)
	n cases	HR	95% CI	HR	95% CI	HR		HR	95% CI	*P* trend	HR	95% CI	
Total cancer	23,142	0.95	0.89, 1.02	0.94	0.88, 1.00	0.94	0.88, 1.01	0.94	0.88, 1.00	0.11	0.98	0.97,1.00	
Lip, oral cavity, pharynx (140–149)	588	1.04	0.67, 1.61	0.93	0.60, 1.44	0.90	0.58, 1.40	1.38	0.91, 2.10	0.18	1.07	0.96, 1.19	
Oesophagus (150)	248	0.95	0.48, 1.86	0.83	0.42, 1.63	0.79	0.40, 1.55	1.12	0.59, 2.12	0.79	0.99	0.84, 1.18	
Stomach (151)	858	0.85	0.60, 1.22	0.78	0.55, 1.11	0.92	0.66, 1.30	0.71	0.50, 1.01	0.14	0.92	0.84, 1.01	
Colon (153)	1,806	0.94	0.72, 1.22	1.19	0.93, 1.53	1.19	0.93, 1.52	1.18	0.92, 1.51	0.04	1.05	0.99, 1.12	
Rectum, anus (154)	1,158	1.12	0.82, 1.53	0.97	0.71, 1.33	0.96	0.70, 1.31	1.09	0.81, 1.48	0.92	1.01	0.93, 1.09	
Liver, intrahepatic bile ducts (155.0)	194	0.33	0.17, 0.63	0.22	0.11, 0.43	0.26	0.13, 0.49	0.14	0.07, 0.29	<0.01	0.58	0.46, 0.71	
Gallbladder, biliary tract (155.1–155.3)	98	1.16	0.38, 3.56	1.35	0.46, 3.98	1.03	0.34, 3.10	1.27	0.44, 3.69	0.80	1.10	0.84, 1.45	
Pancreas (157)	520	0.63	0.41, 0.98	0.75	0.49, 1.14	0.63	0.41, 0.97	0.52	0.33, 0.81	0.01	0.86	0.76, 0.97	
Larynx, trachea/bronchus/lung (161,162)	2,922	1.01	0.83, 1.24	1.09	0.90, 1.33	0.97	0.80, 1.18	1.15	0.95, 1.40	0.22	1.03	0.98, 1.08	
Prostate (177)	6,884	1.00	0.88, 1.14	1.05	0.93, 1.20	1.01	0.89 1.14	0.99	0.88, 1.13	0.86	0.99	0.96, 1.03	
Testis (178)	278	0.80	0.46, 1.39	1.11	0.64, 1.93	1.34	0.76, 2.36	0.81	0.42, 1.56	0.77	0.97	0.81, 1.16	
Kidney, renal cell (180.0–180.9)	691	1.26	0.84, 1.88	0.99	0.66, 1.29	1.23	0.83, 1.83	1.06	0.71, 1.58	0.94	1.00	0.90, 1.11	
Bladder (181)	1,573	1.27	0.97, 1.65	0.90	0.68, 1.18	1.04	0.80 1.35	1.11	0.85, 1.44	0.96	1.01	0.95, 1.09	
Melanoma of skin (190)	1,074	0.87	0.64, 1.16	0.77	0.57, 1.04	0.76	0.56, 1.03	0.82	0.61, 1.11	0.15	0.93	0.85, 1.02	
Non-melanoma of skin (191)	782	0.77	0.54 1.10	0.70	0.49, 1.01	0.79	0.56, 1.13	0.67	0.46, 0.95	0.07	0.91	0.82, 1.01	
Brain, nervous tissue (193)	427	0.91	0.56, 1.47	0.97	0.60, 1.55	0.82	0.50 1.33	0.86	0.53, 1.41	0.48	0.98	0.86, 1.12	
Thyroid gland (194)	128	1.03	0.44, 2.40	0.77	0.32, 1.88	1.01	0.43, 2.40	0.64	0.25, 1.62	0.40	0.94	0.74, 1.21	
Lymph/hematopoietic tissue (200–209)	1,790	0.76	0.60, 0.97	0.75	0.59, 0.95	0.85	0.67, 1.07	0.68	0.54, 0.87	0.02	0.91	0.85, 0.97	
Other cancer	1,123	0.94	0.69, 1.27	0.83	0.61, 1.13	0.85	0.63, 1.15	0.87	0.64, 1.18	0.31	0.96	0.89, 1.05	

Abbreviations: CI, confidence interval; HR, hazard ratio; ICD-7, International Classification of Diseases, seventh revision.

a HRs estimated from Cox proportional hazard regression models with age as the time scale, stratified by cohort, fasting status, and birth year categories, adjusted for baseline age, body mass index categories, and smoking status. HRs corrected for random error by regression dilution ratio (RDR); conversion into uncorrected HR = exp(ln(HR)*RDR), where RDR = 0.644.

**Table 3:** RDR corrected hazard ratios^a^ of incident cancer by cholesterol in quintiles (compared to the lowest quintile) and per unit increment in women.

		Quintile											
Site (ICD-7 code)	n cases	2		3		4		5			per 1 unit		(mmol/l)
		HR	95% CI	HR	95% CI	HR	95% CI	HR	95% CI	*P* trend	HR	95% CI	
Total cancer	15,836	0.95	0.87, 1.03	0.93	0.85, 1.01	0.94	0.86, 1.02	0.86	0.79, 0.93	<0.01	0.95	0.93, 0.97	
Lip, oral cavity, pharynx (140–149)	186	1.19	0.53, 2.68	0.75	0.32, 1.74	1.26	0.58, 2.75	1.13	0.51, 2.48	0.68	1.11	0.92, 1.34	
Oesophagus (150)	47	1.17	0.17, 7.98	1.10	0.17, 7.21	3.29	0.61, 17.81	1.51	0.26, 8.83	0.35	1.02	0.70, 1.49	
Stomach (151)	416	0.96	0.55, 1.68	0.62	0.35, 1.09	0.98	0.57, 1.64	0.84	0.50, 1.42	0.72	0.96	0.85, 1.09	
Colon (153)	1,336	1.05	0.75, 1.48	1.19	0.86, 1.64	1.17	0.85, 1.61	1.23	0.90, 1.69	0.15	1.03	0.96, 1.10	
Rectum, anus (154)	635	1.11	0.68, 1.81	1.43	0.90, 2.26	1.50	0.95, 2.35	1.48	0.94, 2.32	0.49	1.09	0.99, 1.21	
Liver, intrahepatic bile ducts (155.0)	71	2.87	0.69, 11.83	1.08	0.24, 4.86	1.09	0.25, 4.70	0.96	0.22, 4.07	0.24	0.73	0.53, 1.02	
Gallbladder, biliary tract (155.1–155.3)	104	0.94	0.35, 2.51	0.38	0.13, 1.09	0.47	0.18, 1.25	0.23	0.08, 0.62	<0.01	0.66	0.50, 0.86	
Pancreas (157)	324	0.86	0.45, 1.67	0.76	0.40, 1.45	0.97	0.53, 1.80	1.08	0.60, 1.97	0.44	1.06	0.92, 1.23	
Larynx, trachea/bronchus/lung (161,162)	947	0.83	0.56, 1.21	1.10	0.77, 1.57	1.04	0.73, 1.49	1.24	0.88, 1.76	0.05	1.03	0.95, 1.12	
Breast (170)	5,228	0.93	0.81, 1.07	0.91	0.79, 1.04	0.84	0.73, 0.96	0.70	0.61, 0.81	<0.01	0.90	0.86, 0.93	
Cervix (171)	477	1.02	0.66, 1.59	0.97	0.62, 1.52	1.07	0.68, 1.67	1.05	0.66, 1.67	0.79	1.02	0.90, 1.16	
Other parts of uterus (172,174)	1,081	0.83	0.59, 1.16	0.87	0.63, 1.20	0.70	0.51, 0.97	0.74	0.54, 1.02	0.04	0.91	0.84, 0.99	
Ovary (175.0)	733	1.06	0.71, 1.60	1.32	0.89, 1.95	1.42	0.96, 2.09	1.27	0.86, 1.89	0.12	1.06	0.96, 1.17	
Kidney, renal cell (180.0–180.9)	321	0.74	0.37, 1.48	1.12	0.60, 2.11	0.95	0.51, 1.79	1.13	0.61, 2.07	0.40	1.05	0.91, 1.21	
Bladder (181)	325	0.74	0.39, 1.40	0.81	0.44, 1.49	0.70	0.38, 1.28	0.91	0.51, 1.62	1.00	0.95	0.82, 1.10	
Melanoma of skin (190)	777	0.96	0.68, 1.34	0.68	0.48, 0.98	0.98	0.70, 1.38	0.61	0.42, 0.88	0.03	0.88	0.79, 0.98	
Non-melanoma of skin (191)	396	1.67	0.88, 3.16	1.12	0.59, 2.14	1.47	0.80, 2.72	1.52	0.83, 2.78	0.32	1.10	0.97, 1.25	
Brain, nervous tissue (193)	258	0.74	0.40, 1.39	0.56	0.29, 1.08	0.92	0.50, 1.68	0.70	0.37, 1.31	0.54	0.95	0.80, 1.13	
Thyroid gland (194)	259	1.17	0.64, 2.13	0.90	0.48, 1.67	0.93	0.50, 1.72	0.85	0.56, 1.30	0.31	0.88	0.73, 1.05	
Lymph/hematopoietic tissue (200–209)	1,094	0.77	0.56, 1.07	0.81	0.59, 1.11	0.77	0.57, 1.05	0.61	0.44, 0.83	0.01	0.85	0.78, 0.92	
Other cancer	821	1.13	0.77, 1.65	1.10	0.75, 1.60	1.06	0.73, 1.54	0.94	0.64, 1.37	0.51	1.01	0.92, 1.11	

Abbreviations: CI, confidence interval; HR, hazard ratio; ICD-7, International Classification of Diseases, seventh revision.

a HRs estimated from Cox proportional hazard regression models with age as the time scale, stratified by cohort, fasting status, and birth year categories, adjusted for baseline age, body mass index categories, and smoking status. HRs corrected for random error by regression dilution ratio (RDR); conversion into uncorrected HR = exp(ln(HR)*RDR), where RDR = 0.660.

Among men, compared with the first quintile, TSC concentrations in the fifth quintile were borderline significantly associated with decreasing risk of total cancer (HR = 0.94; 95%CI: 0.88, 1.00) and significant inverse associations were observed for cancers of the liver/intrahepatic bile duct (HR = 0.14; 95%CI: 0.07, 0.29), pancreas cancer (HR = 0.52, 95% CI: 0.33, 0.81), non-melanoma of skin (HR = 0.67; 95%CI: 0.46, 0.95), and cancers of the lymph/hematopoietic tissue (HR = 0.68, 95%CI: 0.54, 0.87). Similar associations were observed when one unit increments of TSC were considered (Table 2).

In women, the hazard ratio for the fifth quintile was associated with decreasing risk of total cancer (HR = 0.86, 95%CI: 0.79, 0.93) and furthermore for cancers of the gallbladder (HR = 0.23, 95%CI: 0.08, 0.62), breast (HR = 0.70, 95%CI: 0.61, 0.81), melanoma of skin (HR = 0.61, 95%CI: 0.42, 0.88) and cancers of the lymph−/hematopoietic tissue (HR = 0.61, 95%CI: 0.44, 0.83). Hazard ratios per one unit TSC increment showed similar inverse associations. Additionally, a borderline significant association was observed for cancers of other parts of uterus (HR = 0.91, 95%CI: 0.84, 0.99).

### Lag-time Analysis

Considering males, comparing the fifth to the first quintile in lag-time analyses, after leaving out the first year of follow-up significant inverse associations persisted for cancers of the liver/intrahepatic bile ducts (HR = 0.15, 95%CI: 0.08, 0.31) and pancreas cancer (HR = 0.54, 95%CI: 0.35, 0.85). Furthermore, a borderline positive association for colon cancer (HR = 1.30, 95%CI: 1.01, 1.68) was observed. Leaving out the first five years of follow-up, significant inverse associations were still observed for cancers of the liver/intrahepatic bile ducts (HR = 0.24, 95%CI: 0.11, 0.53), pancreas (HR = 0.48, 95%CI: 0.30, 0.78) and non-melanoma of skin (HR = 0.63, 95%CI: 0.42, 0.94). There was again a positive association of TSC with colon cancer (HR = 1.46, 95%CI: 1.10, 1.92). When the first ten years of follow-up were excluded, only associations with pancreas cancer (HR = 0.50, 95%CI: 0.29, 0.88) non-melanoma of skin (HR = 0.56, 95%CI: 0.36, 0.89) and colon cancer (HR = 1.44, 95%CI: 1.05, 1.98) remained.

In females, all reported associations comparing the fifth to the first quintile persisted, when the first year of follow-up was excluded (total cancer HR = 0.90, 95%CI: 0.83, 0.98; gallbladder HR = 0.25, 95%CI: 0.09, 0.71; breast HR = 0.72, 95%CI: 0.62, 0.83; melanoma of skin HR = 0.60, 95%CI: 0.41, 0.89; cancers of the lymph/hematopoietic tissue HR = 0.65, 95%CI: 0.47, 0.90). When the first 5 years were left out, significant associations with breast cancer (HR = 0.71, 95%CI: 0.60, 0.85) and melanoma of skin (HR = 0.54, 95%CI: 0.33, 0.87) were still observed, which also persisted when the first ten years of follow-up were excluded (breast cancer HR = 0.62, 95%CI: 0.49, 0.80; melanoma of skin HR = 0.46, 95%CI: 0.21, 0.96).

### Sub-analyses before 1994, the Onset of Statin Medication

Results of the sub-analyses including cholesterol data before 1994 are presented in Tables S3 and S4. The results did not show substantial differences in comparison to the main analyses with some exceptions, e.g. the inverse association of TSC with cancer became insignificant for pancreas cancer and cancers of the lymph−/hematopoietic tissue.

## Discussion

In the present prospective cohort study, elevated TSC levels were significantly associated with decreased risk of cancer incidence in general and with several site-specific cancers in men and women. With the exception of male colon cancer we only found no or inverse relationships between TSC and cancer. Inverse relationships were found for cancers of the liver/intrahepatic bile duct, pancreas, non-melanoma of skin and lymph/hematopoietic tissue among men and for gallbladder, breast, melanoma of skin and lymph/hematopoietic tissue among women. From these, only associations of TSC with colon cancer, pancreas cancer, breast cancer, and skin cancer remained significant in the lag-time analysis. Restricting analyses to measurements before 1994, the onset of statin medication, revealed no major differences regarding the estimated associations.

In previous studies, the “preclinical cancer effect” hypothesis [20] has received considerable attention as an explanation for some of the observed inverse associations. That is, the inverse relation between low TSC levels and cancer risk might be caused by cancers in preclinical stages, as malignant neoplasm are known to have protean physiological effect, which might include metabolic depression of blood cholesterol [33]. Additional evidence for reverse causation comes from Trompet et al’s Mendelian randomization study [21] and a review on clinical trials investigating the relationship of low cholesterol and disease activity [34]. Furthermore, it has been suggested that inverse cancer-cholesterol relationships could be explained by competing risks, i.e that patients showing high TSC levels are more likely to be censored due to cardiovascular mortality before they were diagnosed with cancer [20].

Concerning site-specific cancers, reports on associations with colon cancer are controversial. Positive as well as negative associations have been observed [35], [36]. Our results indicate only a modest positive association among men and absence of a relation among women.

Regarding liver cancer, our results are in line with previously published results of the Me-Can study collaboration and other studies, where mostly negative associations have been reported that diminished with increasing lag-time periods [1], [3], [37], [38]. There seems to be a general consensus that, when hepatic inadequacy occurs because of liver cancer and chronic liver disease, form, esterification and evacuation of cholesterol are blocked, which causes changes in cholesterol levels [39].

For gallbladder/biliary tract cancer Andreotti et al [19] reported a U-shaped association, with low levels as well as high levels of cholesterol being linked with excess risks of biliary tract cancers. This was not confirmed in our data; our results showed no significant association in males and a clear inverse association in females.

The amount of literature on pancreatic cancer and its associations with cholesterol is limited. Two conducted studies found no significant associations [40], [41]. Our results differed between males and females with inverse associations in males and non-significant associations in females [42].

With regard to cancers of the lymph/hematopoietic tissue, leukemic blood and bone narrow cells have been reported to show an elevated low density lipoprotein-receptor activity that was inversely associated with plasma cholesterol levels which might explain hypocholesteraemia often seen in leukemic patients [33]. This interpretation is in line with our data, where associations of blood cancer with TSC disappeared in the lag-time analysis.

Most investigations on breast cancer have not reported significant associations with TSC [20], [43], [44]. However, our data showed a clear negative association (see also [45]) that persisted even when the first 10 years of follow-up were excluded, indicating that reverse causation does not apply in this case. Further, Fagherazzi et al [46] found a significantly decreased breast cancer risk among women using cholesterol-lowering drugs. Unfortunately, we do not have any data regarding statin prescription in the Me-Can project to confirm this finding. Associations of TSC with breast cancer were, however, similar in the pre-statin period before 1994 and in the total observation period. Associations of TSC with skin cancer where a debate is going on whether statin use affects skin cancer outcomes [47], [48], were also similar in the two periods.

Recently several authors reported positive associations between TSC levels and aggressive prostate cancer [11]–[13], even when TSC was not associated with overall prostate cancer [11]. Unfortunately, we did not have information regarding prostate cancer grading in our data, so we cannot contribute to this discussion.

Strengths of our study include the large sample size of over 500,000 participants from seven European population-based cohorts with virtually complete capture of cancer cases. We were also able to correct risk estimates for regression dilution bias, caused by random fluctuations in baseline measurements common to long-term prospective studies, which might lead to underestimation of the true risk. Furthermore, all analyses were adjusted for potential confounders such as BMI and smoking status and stratified by birth year, cohort and fasting time before measurement.

On the other hand, our study is limited by the lack of information of use of anti-hypercholesterol medication, such as statins, behavioural aspects like dietary habits, physical activity and alcohol consumption, as well as genetic variations that could have influenced both cholesterol levels and cancer. Furthermore, we did not have separate data on low and high density lipoprotein cholesterol subfractions or detailed information on tumor staging.

In summary, TSC levels were negatively associated with risk of cancer overall in females and risk of cancer at several sites in both males and females. Also, a positive relation was found for colon cancer in men. In the lag-time analysis some associations persisted, suggesting that although competing risks and reverse causation may explain the mainly inverse associations, some etiologic role for this lipid fraction cannot be ruled out.

## Supporting Information

Table S1 **Uncorrected hazard ratios of incident cancer by cholesterol in quintiles (compared to the lowest quintile) and per unit increment in men.**
(DOCX)

Table S2 **Uncorrected hazard ratios of incident cancer by cholesterol in quintiles (compared to the lowest quintile) and per unit increment in women.**
(DOCX)

Table S3 **Uncorrected hazard ratios of incident cancer by cholesterol in quintiles (compared to the lowest quintile) and per unit increment in men, including measurements before 1994.**
(DOCX)

Table S4 **Uncorrected hazard ratios of incident cancer by cholesterol in quintiles (compared to the lowest quintile) and per unit increment in women, including measurements before 1994.**
(DOCX)
