# Supplementary material for: Total Serum Cholesterol and Cancer Incidence in the Metabolic Syndrome and Cancer Project (Me-Can)
Source: PLoS One. 2013 Jan 23;8(1):e54242. doi: 10.1371/journal.pone.0054242 (PMC3553083; doi:10.1371/journal.pone.0054242)
Supplement: Table S2 — Uncorrected hazard ratios of incident cancer by cholesterol in quintiles (compared to the lowest quintile) and per unit increment in women. (DOCX) [file pone.0054242.s002.docx]

Table S2. Uncorrected hazard ratios^a^ of incident cancer by cholesterol in quintiles (compared to the lowest quintile) and per unit increment in women

|  |  | Quintiles | | | | | | | | | | | |  |  | |
| --- | --- | --- | --- | --- | --- | --- | --- | --- | --- | --- | --- | --- | --- | --- | --- | --- |
| Site (ICD-7 code) |  | 2 | |  | 3 | |  | 4 | |  | 5 | |  |  | per 1 unit (mmol/l) | |
|  | n cases | HR | 95% CI | | HR | 95% CI | | HR | 95% CI | | HR | 95% CI | | *P* trend | HR | 95% CI |
|  |  |  |  | |  |  | |  |  | |  |  | |  |  |  |
| Total cancer | 15,836 | 0.96 | 0.91, 1.02 | | 0.95 | 0.90, 1.01 | | 0.96 | 0.91, 1.01 | | 0.90 | 0.86, 0.96 | | <0.01 | 0.97 | 0.96, 0.98 |
| Lip, oral cavity, pharynx (140-149) | 186 | 1.12 | 0.66, 1.92 | | 0.83 | 0.47, 1.44 | | 1.16 | 0.69, 1.95 | | 1.08 | 0.64, 1.82 | | 0.68 | 1.07 | 0.95, 1.21 |
| Oesophagus (150) | 47 | 1.11 | 0.31, 3.94 | | 1.07 | 0.31, 3.68 | | 2.19 | 0.72, 6.69 | | 1.31 | 0.41, 4.21 | | 0.35 | 1.01 | 0.79, 1.30 |
| Stomach (151) | 416 | 0.98 | 0.68, 1.41 | | 0.73 | 0.50, 1.06 | | 0.98 | 0.69, 1.39 | | 0.89 | 0.63, 1.26 | | 0.72 | 0.97 | 0.99, 1.06 |
| Colon (153) | 1,336 | 1.04 | 0.83, 1.30 | | 1.12 | 0.91, 1.39 | | 1.11 | 0.90, 1.37 | | 1.15 | 0.94, 1.41 | | 0.15 | 1.02 | 0.97, 1.07 |
| Rectum, anus (154) | 635 | 1.07 | 0.78, 1.48 | | 1.27 | 0.93, 1.72 | | 1.30 | 0.97, 1.76 | | 1.30 | 0.96, 1.74 | | 0.49 | 1.06 | 0.99, 1.13 |
| Liver, intrahepatic bile ducts (155.0) | 71 | 2.00 | 0.79, 5.11 | | 1.05 | 0.39, 2.84 | | 1.06 | 0.40, 2.78 | | 0.97 | 0.37, 2.53 | | 0.24 | 0.81 | 0.66, 1.01 |
| Gallbladder, biliary tract (155.1-155.3) | 104 | 0.96 | 0.50, 1.83 | | 0.53 | 0.26, 1.06 | | 0.61 | 0.32, 1.16 | | 0.37 | 0.19, 0.73 | | <0.01 | 0.76 | 0.63, 0.91 |
| Pancreas (157) | 324 | 0.91 | 0.59, 1.40 | | 0.84 | 0.55, 1.28 | | 0.98 | 0.66, 1.47 | | 1.05 | 0.71, 1.56 | | 0.44 | 1.04 | 0.95, 1.14 |
| Larynx, trachea/bronchus/lung (161,162) | 947 | 0.88 | 0.69, 1.13 | | 1.06 | 0.84, 1.35 | | 1.03 | 0.81, 1.30 | | 1.16 | 0.92, 1.45 | | 0.05 | 1.02 | 0.96, 1.08 |
| Breast (170) | 5,228 | 0.96 | 0.87, 1.05 | | 0.94 | 0.86, 1.03 | | 0.89 | 0.81, 0.97 | | 0.79 | 0.72, 0.87 | | <0.01 | 0.93 | 0.91, 0.95 |
| Cervix (171) | 477 | 1.01 | 0.76, 1.36 | | 0.98 | 0.81, 1.20 | | 1.04 | 0.73, 1.32 | | 1.03 | 0.76, 1.40 | | 0.79 | 1.01 | 0.93, 1.10 |
| Other parts of uterus (172,174) | 1,081 | 0.88 | 0.71, 1.10 | | 0.91 | 0.74, 1.13 | | 0.79 | 0.64, 0.98 | | 0.82 | 0.67, 1.01 | | 0.04 | 0.94 | 0.89, 0.99 |
| Ovary (175.0) | 733 | 1.04 | 0.79, 1.36 | | 1.20 | 0.92, 1.56 | | 1.26 | 0.97, 1.63 | | 1.17 | 0.90, 1.52 | | 0.12 | 1.04 | 0.98, 1.11 |
| Kidney, renal cell (180.0-180.9) | 321 | 0.82 | 0.52, 1.29 | | 1.08 | 0.71, 1.64 | | 0.97 | 0.64, 1.47 | | 1.08 | 0.72, 1.62 | | 0.40 | 1.03 | 0.94, 1.14 |
| Bladder (181) | 325 | 0.82 | 0.53, 1.25 | | 0.87 | 0.58, 1.30 | | 0.79 | 0.53, 1.18 | | 0.94 | 0.64, 1.38 | | 1.00 | 0.97 | 0.88, 1.07 |
| Melanoma of skin (190) | 777 | 0.97 | 0.78, 1.22 | | 0.78 | 0.62, 0.99 | | 0.99 | 0.79, 1.24 | | 0.72 | 0.56, 0.92 | | 0.03 | 0.92 | 0.86, 0.99 |
| Non-melanoma of skin (191) | 396 | 1.40 | 0.92, 2.14 | | 1.08 | 0.71, 1.65 | | 1.29 | 0.86, 1.93 | | 1.32 | 0.89, 1.96 | | 0.32 | 1.06 | 0.98, 1.16 |
| Brain, nervous tissue (193) | 258 | 0.82 | 0.54, 1.24 | | 0.69 | 0.45, 1.05 | | 0.95 | 0.64, 1.41 | | 0.79 | 0.52, 1.19 | | 0.54 | 0.96 | 0.86, 1.08 |
| Thyroid gland (194) | 259 | 1.11 | 0.75, 1.65 | | 0.93 | 0.62, 1.40 | | 0.95 | 0.63, 1.43 | | 0.85 | 0.56, 1.30 | | 0.31 | 0.92 | 0.82, 1.03 |
| Lymph/hematopoietic tissue (200-209) | 1,094 | 0.84 | 0.68, 1.05 | | 0.87 | 0.71, 1.07 | | 0.84 | 0.69, 1.03 | | 0.72 | 0.59, 0.89 | | 0.01 | 0.90 | 0.85, 0.95 |
| Other cancer | 821 | 1.08 | 0.84, 1.39 | | 1.06 | 0.83, 1.36 | | 1.04 | 0.81, 1.33 | | 0.96 | 0.75, 1.23 | | 0.51 | 1.01 | 0.95, 1.07 |

Abbreviations: CI, confidence interval; HR, hazard ratio; ICD-7, International Classification of Diseases, seventh revision

^a^ HRs estimated from Cox proportional hazard regression models with age as the time scale, stratified by cohort, fasting status, and birth year categories, adjusted for baseline age, body mass index categories, and smoking status.
